# Patterns of Geographic Expansion of *Aedes aegypti* in the Peruvian Amazon

**DOI:** 10.1371/journal.pntd.0003033

**Published:** 2014-08-07

**Authors:** Sarah Anne Guagliardo, José Luis Barboza, Amy C. Morrison, Helvio Astete, Gonzalo Vazquez-Prokopec, Uriel Kitron

**Affiliations:** 1 Department of Environmental Sciences, Emory University, Atlanta, Georgia, United States of America; 2 Universidad Nacional de la Amazonía Peruana, Iquitos, Peru; 3 Department of Entomology, University of California, Davis, Davis, California, United States of America; Naval Medical Research Unit No. 6 (NAMRU-6) Iquitos Laboratory, Iquitos, Peru; 5 Fogarty International Center, National Institutes of Health, Bethesda, Maryland, United States of America; Centers for Disease Control and Prevention, Puerto Rico, United States of America

## Abstract

**Background and Objectives:**

In the Peruvian Amazon, the dengue vector *Aedes aegypti* is abundant in large urban centers such as Iquitos. In recent years, it has also been found in a number of neighboring rural communities with similar climatic and socioeconomic conditions. To better understand *Ae. aegypti* spread, we compared characteristics of communities, houses, and containers in infested and uninfested communities.

**Methods:**

We conducted pupal-demographic surveys and deployed ovitraps in 34 communities surrounding the city of Iquitos. Communities surveyed were located along two transects: the Amazon River and a 95km highway. We calculated entomological indices, mapped *Ae. aegypti* presence, and developed univariable and multivariable logistic regression models to predict *Ae. aegypti* presence at the community, household, or container level.

**Results:**

Large communities closer to Iquitos were more likely to be infested with *Ae. aegypti*. Within infested communities, houses with *Ae. aegypti* had more passively-filled containers and were more often infested with other mosquito genera than houses without *Ae. aegypti*. For containers, large water tanks/drums and containers with solar exposure were more likely to be infested with *Ae. aegypti*. Maps of *Ae. aegypti* presence revealed a linear pattern of infestation along the highway, and a scattered pattern along the Amazon River. We also identified the geographical limit of *Ae. aegypti* expansion along the highway at 19.3 km south of Iquitos.

**Conclusion:**

In the Peruvian Amazon, *Ae. aegypti* geographic spread is driven by human transportation networks along rivers and highways. Our results suggest that urban development and oviposition site availability drive *Ae. aegypti* colonization along roads. Along rivers, boat traffic is likely to drive long-distance dispersal via unintentional transport of mosquitoes on boats.

## Author Summary

*Ae. aegypti* mosquitoes carry a number of viruses that cause human disease, including dengue and yellow fever. Over the past 30 years, the burden of dengue has increased exponentially, due to urbanization, poor waste and water management, human transportation, and expanding mosquito populations. Although much research has been conducted on *Ae. aegypti* at the household and container levels, little is known about the mechanisms fueling the range expansion of this mosquito across longer distances. The goal of this study is to characterize *Ae. aegypti* spread along transportation networks and to identify risk factors associated with its establishment, thus improving our ability to predict future *Ae. aegypti* expansion. Characterizing current patterns of establishment will aid in understanding and preventing future invasions. Our approach is broadly applicable to other biological invasions associated with human activities.

## Introduction

*Aedes aegypti* is the vector of several arboviruses of major global health importance, including dengue virus (DENV), yellow fever virus, and chikungunya and Mayaro viruses. Of these, dengue virus is the most prevalent and geographically extensive, with approximately 2.5 billion people at risk worldwide [1], and 390 million new dengue infections each year [2]. This mosquito vector is well-adapted to the urban environment: females feed almost exclusively on humans, prefer to rest in dark, cool areas (usually indoors) [3], and adult female mosquitoes lay their eggs on the walls of water-filled artificial containers found in and around the home such as vases, plastic buckets, water storage tanks, and discarded refuse and tires [4]. These adaptations to human environments, coupled with the longevity and resistance of its eggs to desiccation [5], [6], contribute to the vector's passive spread to new areas via human transportation networks [7]. In the absence of a vaccine or cure for dengue, most dengue control programs rely on the suppression of vector populations to prevent human exposure to infected mosquitoes. Accordingly, understanding the geographic distribution and range expansion of this vector is of utmost importance for disease surveillance and control.

Originally African in origin, it is thought that *Ae. aegypti* was transported inadvertently to the Americas via European ships used for trade, commerce, and slave transport in the 17^th^–19^th^ centuries [8]. As urbanization continued and the shipping industry expanded, outbreaks of dengue-like illnesses became more common in port cities [8]. By the 20th century, *Ae. aegypti* was present throughout North and South America, probably first infesting port cities and then moving inland [8], [9]. During the mid-20th century (1946-1963), *Ae. aegypti* populations in the Americas were dramatically reduced as a result of a yellow fever control program led by the Pan American Health Organization [10], [11]. The successful reduction of yellow fever led to the waning of control programs targeting the mosquito, and consequentially, since the 1980s, *Ae. aegypti* has become reestablished throughout the Americas [12].

In Latin America and in Peru, *Ae. aegypti* mosquitoes are in the process of expanding from urban to rural areas [13], [14]. The Amazonian city of Iquitos was the first site of *Ae. aegypti* (in 1984) and dengue (in 1990) reestablishment in Peru [15]. Iquitos rests at the intersection of the Amazon, Nanay, and Itaya Rivers which connect it to a number of smaller settlements throughout the region. Despite apparently similar climatic and socioeconomic conditions shared by most communities in the region, *Ae. aegypti* is heterogeneously distributed among these rural settlements.

Invasion ecologists describe the invasion process as a series of sequential steps that include transport to a new area, release, establishment, and spread [16]. Invasion success is determined by several factors including the number and frequency of introduction events (propagule pressure) [17], [18], abiotic and biotic properties of the receiving ecosystem [19]–[22], and behavior of the invader (e.g., tolerance of or attraction to human environments, oviposition behavior) [16]. In this study, we focus on introductory pressure (by measuring the number of vehicles and trips traveling to and from each town), selected abiotic factors (e.g., container abundance), and biotic factors (e.g., presence of other mosquito species). Understanding the mechanisms underlying these invasion steps for *Ae. aegypti* is critical for predicting and mitigating future expansion of this mosquito species and the pathogens it transmits.

The region surrounding Iquitos provides an ideal setting to study these questions, due to the presence of an interconnected network of settlements with different population sizes, varying degrees of urbanization, reliance on both river and road transit, and similar climatic conditions (e.g., temperature, rainfall) at this ecological scale. In this context, we used two pupal-demographic datasets (an existing historical dataset from 2008-11 and more in-depth data collected for this study from 2011-12) to address two questions: 1) Which factors are associated with *Ae. aegypti* establishment in rural areas?; and 2) How do different human transportation networks influence *Ae. aegypti* spread (rivers vs. roads, primary vs. secondary transportation routes)?

## Methods

### Ethics Statement

No personal information was collected during interviews. Permission for this study was granted by the Loreto Regional Health Department, and the study protocol was approved by the NAMRU-6 Institutional Review Board in compliance with all applicable federal regulations governing the protection of human subjects (protocol number NAMRU6.2012.0039). In addition, the Emory University Institutional Review Board determined that this study does not represent human subjects based research.

### Study Area

**Figure 1:**
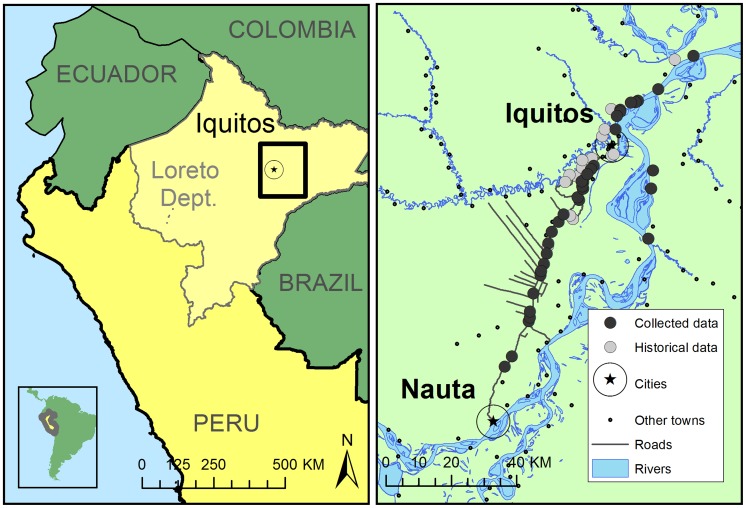
Map of study area. Iquitos is the largest city in the Peruvian Amazon (pop: 380,000), and is accessible only by boat or plane. There are approximately 500,000 people living in the study area shown on the right side of the map. Fluvial routes are the predominant mode of transportation in the region.

With approximately 380,000 inhabitants, Iquitos is the largest population center in the Department of Loreto, Peru. Transportation pathways, including new roads and river routes have been developed over the course of the past 30 years as a result of increased commerce and trade in natural resources (e.g., oil, timber, and coca). As a result, small settlements in the region have experienced rapid population growth and expansion [23]. Although river networks are the predominant mode of transit, a 95 km road connecting Iquitos to the smaller city of Nauta (population: 17,000) facilitates terrestrial commerce and population movement. With its construction, the road has brought about the establishment of new settlements in areas that were previously inaccessible, human population growth (growth rate approximately 4% greater than that of Iquitos), and deforestation due to farming [24]. Communities included in the present study (**Table S1**) can largely be described as “rural” due to small population sizes (ranging from ∼100 to 6,000 inhabitants), geographic isolation from large cities, and limited access to cellular networks and other communication channels. People living in these communities subsist on hunting, fishing, and small-scale agriculture, such as cassava root and plantain farming [25]. Water is derived from a variety of sources: piped water systems (accessible in the home via a faucet but not potable), water collected in buckets directly from the river, rain water that is actively gathered in large drums from roofs either directly or from gutters, and well water from ground sources. Unmanaged or discarded containers may also be passively filled through the unintentional accumulation of rain water. A map of the study area is shown in **Figure 1**.

### Datasets

We used historical data available from the Peruvian Ministry of Health (MOH) and Naval Medical Research Unit No. 6 (NAMRU-6) to characterize patterns of *Ae. aegypti* expansion at the community scale. NAMRU-6 personnel conducted *Ae. aegypti* pupal-demographic surveys as part of epidemiological studies on alphaviruses, while the Peruvian MOH independently carried out larval surveys as a component of normal surveillance activities. The MOH and NAMRU-6 data consisted of information about 31 communities and two cities in the region during 2008, 2011, and 2012, and were obtained by surveying approximately 10% of houses in a community [26]. Each house was searched thoroughly for *Ae. aegypti* mosquito larvae and pupae. In addition, in NAMRU-6 surveys Prokopack aspirators were used to collect adult mosquitoes [27].

To supplement these historical data we selected communities for a more detailed analysis of *Ae. aegypti* presence. To determine mosquito presence in 2011-12, we deployed ovitraps and simultaneously conducted a thorough survey of wet containers (with water at the time they were surveyed) within households (described in further detail below). Communities were selected along two transects: one following the Iquitos- Nauta highway (N = 22) and the other along the Amazon River (N = 12), for a total of 34 communities. The geographic limit of transects was defined by the network distance (travel time) from Iquitos, under the assumption that long-distance dispersal of *Ae. aegypti* is due to unintentional passive human transport of immature and adult mosquitoes. For the Iquitos-Nauta highway, two hours of travel time resulted in a path-distance from Iquitos of 76.4 km. For the Amazon River, two hours of travel time in the fastest vehicle (a speed boat) translated into an approximate 44.5 km path-distance from Iquitos.

In all, we collected information about 48 rural communities (**Table S1**). Iquitos and Nauta were excluded from all analyses, as the purpose of this study was to understand *Ae. aegypti* expansion from urban to peri-urban and rural areas. We also collected information on the year of community incorporation, water system type, the number of inhabitants, and the number of houses (obtained from the 2007 Peruvian National Census) [28]. When census data were not available, population estimates were obtained from local authorities such as the mayor or the health center director.

With the exception of one site, El Terminal, all of the study sites are towns that have been officially incorporated. While El Terminal (a bus station and residential area) is not politically separated from Iquitos, it is geographically far enough to be ecologically distinct. (The distance between Iquitos and El Terminal, ∼400 m, exceeds that of the estimated *Ae. aegypti* flight range, ∼100 m [29]-[32].)

### Entomological Data Collection

Houses were systematically selected for *Ae. aegypti* sampling and ovitrap deployment: starting at a randomly selected household within the areas of highest housing density, every Nth house was sampled based on the total number of houses in the community to ensure a minimum coverage of 10%. This resulted in a minimum of 10 and a maximum of 78 houses sampled per community.

During October through December of 2012, ovitraps were deployed in all communities along our transects. Ovitraps were red plastic cups filled ¾ water (volume = 56.5 in^3^) and lined with paper. Two ovitraps were placed within each home in a dark, secluded area where they would not be a nuisance to residents. Eight days after deployment, ovitraps were checked for immature mosquitoes and removed from the household. Immature mosquitoes were collected in sterile bags (Whirlpak Co.) and transported to the field laboratory for rearing and taxonomic identification to species for *Ae. aegypti* and to genus for other mosquitoes. Paper from the ovitraps was thoroughly examined under a microscope to count the number of eggs present. *Ae. aegypti* eggs are easily differentiated from other container-breeding mosquitoes due to their smooth texture, black color, and position above the water line along the sides of containers. There are no other common container breeding *Aedes* species in this region. Although a number of *Ochleratatus* (formerly *Aedes*-genus mosquitoes) have been documented in the area [33], [34], these mosquitoes predominate in natural water bodies in forested areas such as rain pools and swamps [34]–[38]. *Culex* genus mosquitoes are often found in containers but they lay their eggs in rafts on the water surface. Simultaneous with ovitrap deployment, household pupal-demographic surveys were conducted to determine the abundance of wet containers and the presence of other mosquito genera in each household. Wet containers within and around each household were exhaustively surveyed for the presence of mosquito larvae. Using previously established protocols for *Ae. aegypti* surveys in Iquitos, we recorded the following information for each container: container type and material, observed solar exposure (if the container was exposed to direct sunlight at any time during the day; yes/no), degree of organic material present in the water (ranked on a scale of one to three), container location, whether the container was inside or outside or underneath a roof, whether the container was filled manually (including active collection of rain water) or passively (through unintentional rain water accumulation), and the presence of abate larvacide [39], [40]. Mosquito eggs, larvae, and pupae were collected in Whirlpak bags and were transported to the field laboratory for rearing, counting, and taxonomic identification to species for *Ae. aegypti* and to genus for other mosquitoes.

### Transportation Data

River networks are the primary mode of transportation in the region (**Figure 1**). There are a variety of boat types that carry both passengers and cargo throughout the Peruvian Amazon including; large barges (for cargo and passengers), medium-sized barges, speed boats, and small water taxis. Terrestrial vehicle types include mini-buses taxis that travel the Iquitos-Nauta highway.

To characterize the connectivity between Iquitos and surrounding communities, 140 vehicle drivers were interviewed across 11 sites throughout Iquitos: 9 different ports and 2 bus/taxi departure points. Each sampling location was visited twice, and route information was collected for 8 taxis, 14 mini-buses, 19 medium-sized barges, 22 large barges, 25 speed boats, and 52 water taxis (a total of 140 vehicles). All available vehicle drivers were interviewed at each port or bus station. For each vehicle we collected information on the frequency and duration of travel, the final destination of the vehicle, and the number of trips to each community per month.

### Data Management and Analysis

All data analysis and graphs were produced using R statistical software [41]. GPS coordinates of each town were recorded with a Garmin GPSMAP 62sc and integrated with other information (rivers, political boundaries) to create study area maps in ArcMap 10.1 (ESRI, Redlands, CA). Finalized maps were projected in Universal Transverse Mercator (UTM), Zone 18S, WGS1984 datum.

### Community-Level Data

Descriptive maps of *Ae. aegypti* presence were developed by year (2008 and 2011 for the historical data, and 2011-12 for the data collected for this study). (**Table S2** shows datasets and data analyses employed.) A community was considered positive for *Ae. aegypti* if the mosquito was found either via ovitraps or larval surveys. For the data collected for this study (2011-12), we compared median values for community age, number of inhabitants, number of houses, and distance from the city of Iquitos (assumed to be the source population) between positive and negative communities using nonparametric Mann-Whitney Wilcoxon non-paired tests. We measured both Euclidean distance and path-distance – the latter was calculated by tracing the shortest routes from Iquitos (fluvial, terrestrial, or some combination of the two). We calculated the following entomological indices in 34 communities surveyed; Container Index (positive containers/containers surveyed *100), House Index (positive houses/houses surveyed*100), and Breteau Index (positive containers/houses surveyed *100). For one community (Varillal), we found a positive ovitrap but no positive containers from the pupal surveys.

Logistic regression models were used to explore factors associated with *Ae. aegypti* presence. The variable population size was log-transformed to force normality prior to its inclusion in the models. Other variables tested included the total wet containers and passively-filled containers, the average number of wet and passively-filled containers per house, access to the highway vs. the river, water system type, community age, the number of vehicles traveling to each location, the number of high-risk vehicles traveling to each location, and the presence of other mosquito genera. “High risk” vehicles were defined as vehicles that were most likely to contain *Ae. aegypti* mosquitoes, including large river boats for riverine communities and buses for communities along the highway. All possible combinations of variables were explored in each of the models, and the final model was selected using backwards stepwise selection in the MASS package in R [41], [42], based on the Akaike Information Criterion. Only explanatory variables that were significant univariable predictors (p<0.10) were included in the multivariable model. The independence of predictor variables was evaluated by testing regression models of all possible combinations of predictor variables. Residual plots were used to evaluate heteroscedasticity.

### Household-Level Data

For the household-level analysis, we explored variables thought to be predictive of *Ae. aegypti* presence/absence in univariable and multivariable logistic regression models. Predictor variables included; number of people per household, the number of wet and passively-filled containers, the presence of mosquito genera within the house, and the abundance of other mosquito genera within the house.

Multivariable logistic models were developed using backwards stepwise selection in the MASS package in R [42]. The independence of predictor variables was evaluated by running regressions of all possible combinations of predictor variables.

### Container-Level Data

We calculated the proportion of positive containers by container type for each community positive for *Ae. aegypti* mosquitoes. To assess container productivity we calculated the proportion of pupae produced by each container type.

Univariable and multivariable logistic regression models predicting *Ae. aegypti* presence/absence were developed using the following predictor variables; container material (plastic, metal), container type (plastic bucket/pans, large drums and tanks), solar exposure (yes/no), presence or absence of a container cover, presence of other mosquito genera within the same container, the number of other mosquitoes within the same container, and fill method (manually filled vs. passively filled).

A multivariable model was developed using backwards stepwise selection in the MASS package in R [42]. Predictor variables that were correlated with one another were not included in the selection process.

## Results

### Community-Level Analysis

**Figure 2:**
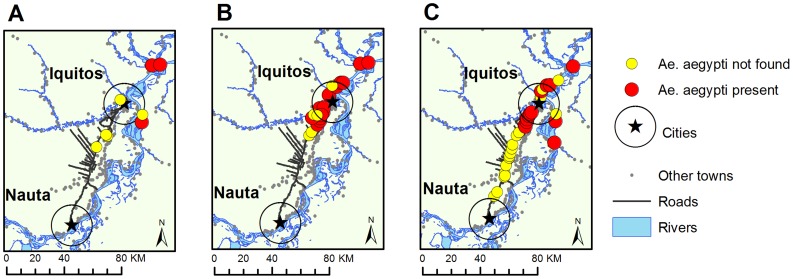
*Ae. aegypti* presence-absence by data source. Results from pupal demographic surveys/ovitraps are shown for A) Historical data from MOH/NAMRU data in 2008, B) Historical data from MOH/NAMRU in 2011, and C) Data collected for this study in 2011-12 (N = 34).

*Ae. aegypti* was the most abundant species, followed by *Culex*-genus mosquitoes. Other mosquito genera included; *Culex*, *Limatus*, *Toxorhynchites*, *Wyeomyia*, *Trichoprosopon*, all of which have been previously reported from the Peruvian Amazon [33], [34]. Among the 34 communities where entomological surveys were conducted for this study, 14 were positive for *Ae. aegypti* (**Figure 2**). Both *Ae. aegypti* and *Culex*-genus mosquitoes were found in 23.5% (8 of the 34 communities surveyed). In 17.6% (6/34) communities *Ae. aegypti* mosquitoes were found without *Culex*, and in 20.6% (7/34) of communities *Culex* mosquitoes were found without *Ae. aegypti*.

We report here the presence of *Ae. aegypti* in three new communities, two along the highway, and one along the Amazon River. Descriptive maps of *Ae. aegypti* infestation based on MOH/NAMRU collections from 2011 showed clustering of *Ae. aegypti* positive towns near Iquitos (**Figure 2**). Data collected for this study in 2011-12, however, showed a clear limit of *Ae. aegypti* expansion along the Iquitos-Nauta highway (Euclidean distance to Iquitos of 19.3 km). River communities, in contrast, showed a more heterogeneous spatial pattern, and the farthest point of expansion from Iquitos was 37.1 km. *Ae. aegypti* entomological indices revealed differences in mosquito abundance across sites (**Table 1**).

**Table 1:** Entomological indices for communities positive for *Ae. aegypti* (collected data only).

Community	Container Index (CI)			House Index (HI)			Breteau Index (BI)		
	Positive containers	Containers surveyed	CI	Positive houses	Houses surveyed	HI	Positive containers	Houses surveyed	BI
Aucayo	27	110	**24.55**	12	15	**80.00**	27	15	**180.00**
Nueva Unión	11	73	**15.07**	6	10	**60.00**	11	10	**110.00**
25 de Enero	6	88	**6.82**	4	11	**36.36**	6	11	**54.55**
Peña Negra	8	157	**5.10**	5	14	**35.71**	8	14	**57.14**
Barrio Florida	7	141	**4.96**	5	16	**31.25**	7	16	**43.75**
Cruz del Sur	3	63	**4.76**	3	13	**23.08**	3	13	**23.08**
Los Delfines	17	331	**5.14**	10	45	**22.22**	17	45	**37.78**
El Terminal	2	73	**2.74**	2	10	**20.00**	2	10	**20.00**
Quistococha	16	338	**4.73**	8	41	**19.51**	16	41	**39.02**
Tamshiyacu	17	556	**3.06**	12	78	**15.38**	17	78	**21.79**
Indiana	13	909	**1.43**	10	76	**13.16**	13	76	**17.11**
Santa Clotilde	1	77	**1.30**	1	9	**11.11**	1	9	**11.11**
5 de Abril	1	60	**1.67**	1	11	**9.09**	1	11	**9.09**

Indices quantifying *Ae. aegypti* densities include; Container Index (positive containers/containers surveyed *100), House Index (positive houses/houses surveyed*100), and Breteau Index (positive containers/houses surveyed *100).

**Figure 3:**
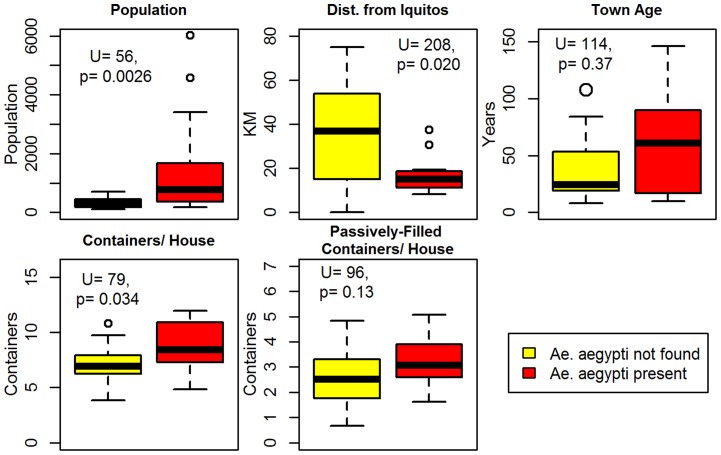
Mann-Whitney Wilcoxon tests for median differences in *Ae. aegypti* positive vs. negative communities. Significant differences (p<0.05) between *Ae. aegypti* positive vs. negative communities were detected in terms of human population size, distance from Iquitos, and the number of wet containers per house.

*Ae. aegypti* positive communities had larger population size (Mann-Whitney, U = 56, p<0.05), were closer to Iquitos (U = 208, p<0.02), and had more wet containers per household (U = 79, p<0.05) than *Ae. aegypti* negative communities (**Figure 3**). No significant differences were detected in terms of community age (U = 114, p>0.5) or the average number of passively-filled containers per household (U = 96, p>0.1).

Univariable logistic regression models (**Table S3)** showed that increased human population (odds ratio, OR = 1.004, p<0.05) and log human population (OR = 5.06, p<0.01) increased the odds of *Ae. aegypti* establishment. Increased Euclidean and path distance from Iquitos were both negatively associated with *Ae. aegypti* presence (OR = 0.94, p<0.05 for both distance measures). A higher number of wet containers resulted in an increased probability of *Ae. aegypti* establishment in that community (OR = 1.03, p<0.05). Since the number of wet containers per community is positively correlated with the population size (R^2^ = 0.67, p<0.0001), we also used the average number of wet containers per household as a predictor variable. A greater number of wet containers per household increased the risk of *Ae. aegypti* establishment by 1.55 times (p<0.05). Communities relying predominantly on river/stream water were much less likely (0.18 times) to have *Ae. aegypti* mosquitoes than those relying on other water sources (e.g., well water, piped water) (p<0.05). Variables with no significant impact on *Ae. aegypti* presence included; community age, the number of vehicles (and high risk vehicles) traveling to each site per month, the average number of passively-filled containers/household, the absolute number of passively-filled containers per household, use of piped water, access to the Amazon River, and presence of other mosquito genera.

For the multivariable model the log-transformed human population number (which had a more powerful effect than the raw population number) was used. Euclidean distance was used because it had a lower AIC value than path distance from Iquitos. The predictor variables included in the model selection process were; log human population, Euclidean distance from Iquitos, the average number of wet containers per household, and whether or not the community relied on river or stream water. The final multivariable logistic regression model (**Table 2**) showed that the risk of *Ae. aegypti* establishment is increased 5.76 times per log population unit (p<0.05), when taking into account the Euclidean distance from Iquitos and the use of river/stream water. Communities farther away from Iquitos were less likely to have *Ae. aegypti* mosquitoes (OR = 0.89, p<0.05) when adjusting for the other variables included in the model. The use of river/stream water was not statistically significant.

**Table 2:** Multivariable logistic regressions: *Ae. aegypti* risk factors at the community scale.

Variable	OR	95% CI	SE	P
**Log(Population)**	**5.76**	**1.78, 34.49**	**0.72**	**<0.05**
**Euclidean dist. from IQT (km)**	**0.89**	**0.76, 0.97**	**0.058**	**<0.05**
River/stream water	0.094	0.0046, 0.90	1.30	>0.05

Statistically significant (p<0.05) variables are shown in bold (N = 34 communities).

### House-Level Analysis

Of the 580 houses (in 34 communities) that we surveyed, 80 (13.8%) were positive for *Ae. aegypti*. Among houses (N = 380) in the 14 positive communities, 22.9% houses were positive (80/350 houses). House-level logistic regression models were restricted to communities in which *Ae. aegypti* was present. In houses in positive communities, *Culex* mosquitoes were found together with *Ae. Aegypti* in 4.2% (16/380) of houses. In 16.6% (63/380) of houses in positive communities, *Ae. aegypti* were found without *Culex*, and in 2.6% (10/380) houses *Culex* mosquitoes were present without *Ae. aegypti*.

Univariable logistic regression (**Table S4**) showed that the number of wet containers found within a household slightly increased the risk of *Ae. aegypti* presence (OR = 1.05, p<0.05). Houses with higher number of passively-filled containers were 1.17 times more likely to have *Ae. aegypti* mosquitoes (p<0.001). Both the presence and number of other mosquitoes within the household increased the risk of *Ae. aegypti* presence (OR = 5.85, p<0.001, and OR = 5.44, p <0.001, respectively).

Given that the numbers of wet and passively-filled containers in a house are correlated, we chose to include only the number of passively-filled containers in the multivariable model, as the AIC and p-value were both lower. Similarly, the number of other mosquitoes was chosen over the presence/absence of other mosquitoes, based on the AIC and significance levels of the univariable predictors. The final multivariable logistic regression model (**Table 3**) showed that the number of passively-filled containers increased risk of *Ae. aegypti* presence 1.16 times (p<0.001), while the presence mosquitoes of other genera present increased this risk 5.67 times (p<0.001).

**Table 3:** Multivariable logistic regressions: *Ae. aegypti* risk factors at the house scale.

Variable	OR	95% CI	SE	P
No. passively-filled containers	1.16	1.08, 1.27	0.04	<0.001
Presence of mosquitoes of other genera	5.67	2.54, 13.05	0.41	<0.001

Statistically significant (p<0.05) variables are shown in bold (N = 380 houses).

### Container-Level Analysis

**Figure 4:**
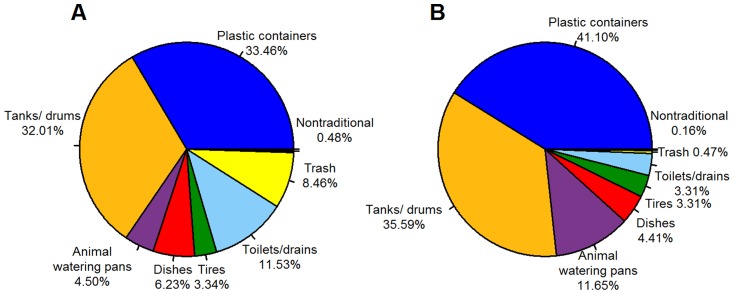
Larval and pupal productivity by container type. A) Larval productivity, B) Pupal productivity.

Among containers that were positive for *Ae. aegypti*, *Culex* genus mosquitoes were also found in 8.6% of containers (11/128). The most common types of water-holding containers found (regardless of infestation status) were plastic buckets (61.2% of all containers) and large water drums (10.5% of all containers). Although plastic containers were very common (1,977 found), the proportion infested was small (2.7%). Toilets and drains had the highest infestation level (17.0% positive of 23), followed by tires (12.8% positive of 39) and large water storage tanks/drums (11.5% positive of 340) (**Table 4**). Productivity analysis by container type (**Figure 4**) demonstrated that plastic containers and water storage tanks/drums produced 41.1% and 35.6% of all pupae, respectively, followed by animal watering pans(11.7%). A similar pattern held for larval productivity, with plastic containers and water tanks/drums accounting for 33.4% and 32.0% of the larvae, respectively.

**Table 4:** Proportion of positive containers by type.

Container Type	Number positive	Number negative	Total Found	Percent Positive
Toilet/drains	4	19	23	17.39
Tires	5	34	39	12.82
Water storage tanks/drums	39	301	340	11.47
Animal watering pans/fish ponds	7	103	110	6.36
Nontraditional (puddles, plants)	3	45	48	6.25
Trash (discarded items)	11	342	353	3.12
Plastic containers (buckets, pans)	54	1923	1977	2.73
Dishes (plates, mugs, plate holders)	5	310	315	1.59
Wells	0	28	28	0.00
**Total**	**128**	**3105**	**3233**	**3.96**

A container was considered positive for *Ae. aegypti* mosquitoes if it had larvae or pupae. (The table does not include two containers that had eggs, but no larvae or pupae.).

Univariable logistic regressions (**Table S5)** showed that toilets/drains (OR = 5.24, p<0.01) and tires (OR = 3.64, p<0.01) were more likely to be infested with *Ae. aegypti* in comparison to other container types. Large water storage tanks and drums increased the probability of *Ae. aegypti* presence 4.04 times (p<0.001), as did the presence of other mosquito genera (OR = 12.60, p<0.001). Containers with solar exposure were more likely to contain *Ae. aegypti* (OR = 2.42, p<0.001). Containers with lids were 0.15 times less likely to have *Ae. aegypti* in comparison with containers without lids (p<0.01). Plastic containers were less likely to be infested with *Ae. aegypti* (OR = 0.44, p<0.001), consistent with the observation that only 2.8% were positive for *Ae. aegypti* even though they comprise the most prevalent container type.

Our multivariable model (**Table** **5**) showed that drums/tanks increased the risk of *Ae. aegypti* infestation by 4.22 times (p<0.0001), while solar exposure increased this risk by 2.53 times (p<0.0001). Because regressions of all possible combinations of predictor variables (**Table S6**) revealed that container type categories were correlated with one another, we used only the value of type =  drum/tank which had the lowest AIC among the univariable models. We also excluded presence/absence of container lids from the final multivariable model because this variable was correlated with both solar exposure and type =  drum/tank.

**Table:** **Table 5.** Multivariable logistic regressions: *Ae. aegypti* risk factors at the container scale.

Variable	OR	95% CI	SE	P
Type = drum/tank	4.22	2.81, 6.23	0.20	<0.0001
Solar Exposure = yes	2.53	1.72, 3.81	0.20	<0.0001

Variables included in the multivariable model selection processes were; type =  drums/tanks, presence of mosquitoes of other genera, solar exposure, and presence of a container lid. Statistically significant (p<0.05) variables are shown in bold (N = 3235 containers).

## Discussion

To our knowledge this is the first extensive, multi-scale analysis of *Ae. aegypti* geographic expansion from urban to peri-urban and rural areas. Of the three ecological scales, the most novel findings were based on our community-level data, as the house and container-level data simply confirmed previous findings about *Ae. aegypti*. At the container-level, for example, water tanks/drums have previously been shown to be important for *Ae. aegypti* production, and *Ae. aegypti* has been shown to co-exist with *Culex* genus mosquitoes [40]. (The co-occurrence of *Ae. aegypti* and *Culex* mosquitoes in households is likely attributable to the abundance of suitable containers that are favorable to all container-breeding mosquitoes, and the availability of shade and sufficient organic material for larval feeding.) Below we discuss further these effects of water use, population size, and distance from Iquitos on the invasion process. We also explore the spatial pattern of *Ae. aegypti* spread, and its implications for DENV transmission.

Community use of river/stream water reduced the odds of *Ae. aegypti* establishment. While this variable was not statistically significant in our multivariable model, we suspect that this may be due to relatively small number of observations and low statistical power. The observed association may be a result one of three mechanisms 1) Water use may be correlated with other factors important for *Ae. aegypti* establishment and spread. (For example, piped water systems are likely to be most abundant in larger settlements closer to Iquitos city.) 2) River/stream water may be less attractive to *Ae. aegypti* mosquitoes for oviposition due to the chemical and organic composition of the water. 3) Containers filled with river/stream water may be frequently emptied and re-filled, thus reducing the probability of the accumulation of organic material and therefore oviposition. For the first mechanism, we were unable to identify a significant correlation between river/stream water usage and population size or distance to Iquitos (**Figure S1**). The second mechanism could be evaluated through simple oviposition experiments to test the hypothesis that river water is less suitable for *Ae. aegypti* oviposition and development, similar to that in [43], [44]. Lastly, longitudinal studies could elucidate patterns of container use and quantify water turnover [45] by water source in rural areas, although this is likely to vary depending on local cultural and socioeconomic conditions.

Human population size may influence *Ae. aegypti* invasion in two ways. First, large population centers are more likely to have an abundance of oviposition sites, thus contributing to greater habitat suitability. Secondly, population centers are also more “connected” to other places via vehicle traffic, thereby contributing to human-mediated introduction of immature mosquitoes (introductory pressure). Thus, from an invasion ecology perspective, we cannot disentangle the effects of habitat suitability vs. introductory pressure, since both are correlated with population size. Regardless of which of these individual mechanisms (or combination of the two) is driving the observed associations, *Ae. aegypti,* control programs should address both habitat suitability (wet container management/reduction, insecticide spraying) and introductory pressure (surveillance and control of mosquitoes on vehicles).

Our analyses revealed that population and inverse distance increased risk of *Ae. aegypti* presence at a community scale. While beyond the scope of this paper, gravity models may be an appropriate way to predict the future spread of *Ae. aegypti* mosquitoes. Although gravity models have been used to model DENV dispersal [46], [47], to date, such an approach has not been applied to *Ae. aegypti* spread. Gravity models assume that connectivity between locations is a function of the inverse distance between them and of their ‘attractiveness’ based on population size. They have been applied to invasive organisms that spread through human-mediated activities [48]–[50]. A key advantage to this approach is that the required data are often easily accessible through public resources such as census data and public maps.

**Figure 5:**
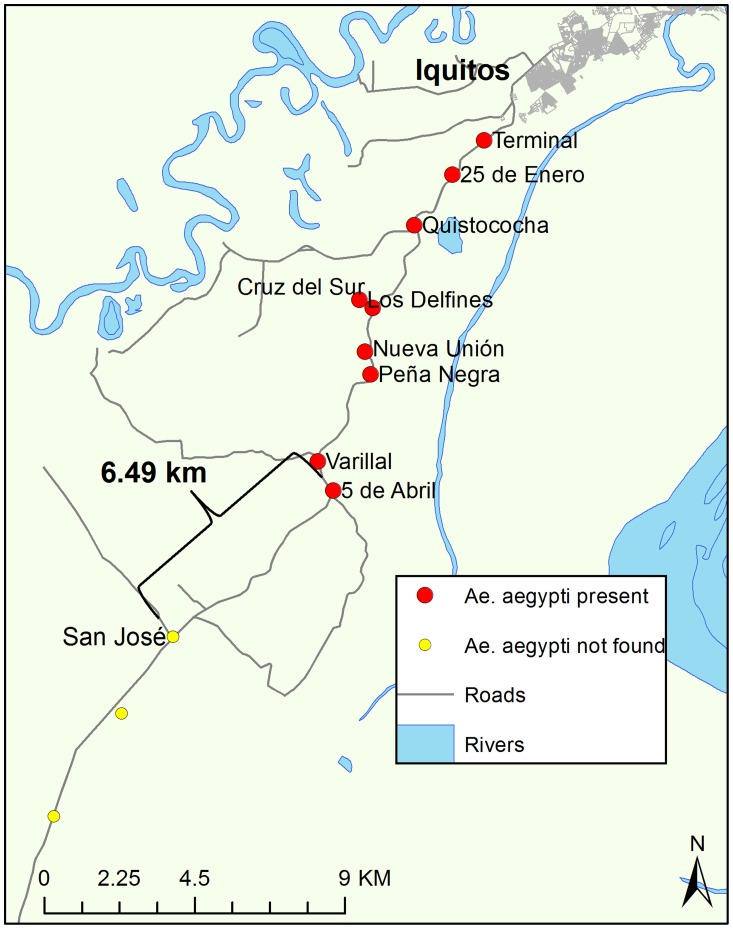
Geographic border of *Ae. aegypti* colonization along the Iquitos-Nauta highway. The distance between the southernmost positive community, 5 de Abril, and the next community, San José, is approximately 6.49 km. The space between these two communities is characterized by forest cover with no human settlements (and therefore no oviposition sites). This forested area likely acts as a barrier to *Ae. aegypti* dispersal.

The contrast between the spatial pattern of *Ae. aegypti* establishment along rivers vs. the highway is noteworthy. Genetic studies have suggested that *Ae. aegypti* spreads along transportation networks [51]–[53], but few studies to date have used field collections to identify areas where *Ae. aegypti* has become successfully established following introduction from a known source [13], [14], [54]. We propose that urbanization is responsible for the linear pattern observed along the highway, due to the high density of settlements relatively close to Iquitos and immediately adjacent to the highway. Short-distance active dispersal of *Ae. aegypti* mosquitoes is driven by availability of oviposition sites [32], and as urbanization continues southward, ovisposition sites become more abundant. The community 5 de Abril represents the geographic limit of *Ae. aegypti* along the highway, approximately 19 km south of Iquitos. This is most likely due to the ∼6.5 km gap between 5 de Abril and the next community to the south, San José (**Figure 5**). Prior to that point, each community is distanced <3.1 km from the next settlement along the highway.

In contrast, it is probable that *Ae. aegypti* geographic expansion along fluvial routes is the result of longer-distance dispersal events that are mediated via the passive transport of mosquito eggs through human vehicles (boats). Urbanization along the highway is more relevant for DENV transmission, owing to greater density of human host and vector populations [55], [56]. Mahabir et al (2012) showed that arterial highways in Trinidad tended to have fewer dengue cases than did smaller, rural roads, as major highways may serve as barriers to transmission [57]. With only one lane in each direction, the Iquitos-Nauta highway would likely be classified as a smaller road leading to rural towns. Thus, we believe that as urbanization south of Iquitos continues, the conditions along the Iquitos-Nauta highway will grow increasingly suitable for DENV transmission. In contrast, while riverine communities may be susceptible to mosquito introductions, most of these towns currently lack enough human hosts to ensure sustained local DENV transmission. (Realistic estimates of minimum human population required for local transmission range from 3,000 to 100,000 [58]–[61].)

Our approach may be applicable to *Ae. aegypti* in other regions (e.g., Vietnam's Mekong Delta which relies heavily on fluvial transport), and to other insect vectors that are passively transported by humans (e.g., *Aedes albopictus* [62], *Culex quinquefasciatus* [63]; and *Triatoma infestans* [64], among others [65]).

## Supporting Information

Figure S1 **River/stream water vs. other water types by population and distance from Iquitos.** Mann-Whitney Wilcoxon tests showed no significant correlation between river/stream water usage and population size or distance to Iquitos.
(TIF)

Table S1 **Characteristics of communities included in the study**. + indicates that *Ae. aegypti* was found, - indicates that *Ae. aegypti* was not found. A blank space indicates that data were not collected for that community.
(DOCX)

Table S2 **Datasets, ecological scales, and statistical analyses employed.**
(DOCX)

Table S3 **Community-level univariable logistic regression models.** Statistically significant (p<0.05) variables are shown in bold. Variables were included in the multivariate selection process with an entry criterion of p<0.10.
(DOCX)

Table S4 **House-level univariable logistic regression models.** Statistically significant (p<0.05) variables are shown in bold. Variables were included in the multivariate selection process with an entry criterion of p<0.10.
(DOCX)

Table S5 **Container-level univariable logistic regression models.** Statistically significant (p<0.05) variables are shown in bold. Variables were included in the multivariate selection process with an entry criterion of p<0.10.
(DOCX)

Table S6 **Container-level univariable logistic regression models demonstrating multicollinearity among predictor variables.** The tables below demonstrate logistic regression models using all possible combinations of predictor variables. Significant (p<0.05) predictor variables are shown in bold.
(DOCX)
