# Supplementary figures and images for: Patterns of Geographic Expansion of Aedes aegypti in the Peruvian Amazon
Source: PLoS Negl Trop Dis. 2014 Aug 7;8(8):e3033. doi: 10.1371/journal.pntd.0003033 (PMC4125293; doi:10.1371/journal.pntd.0003033)

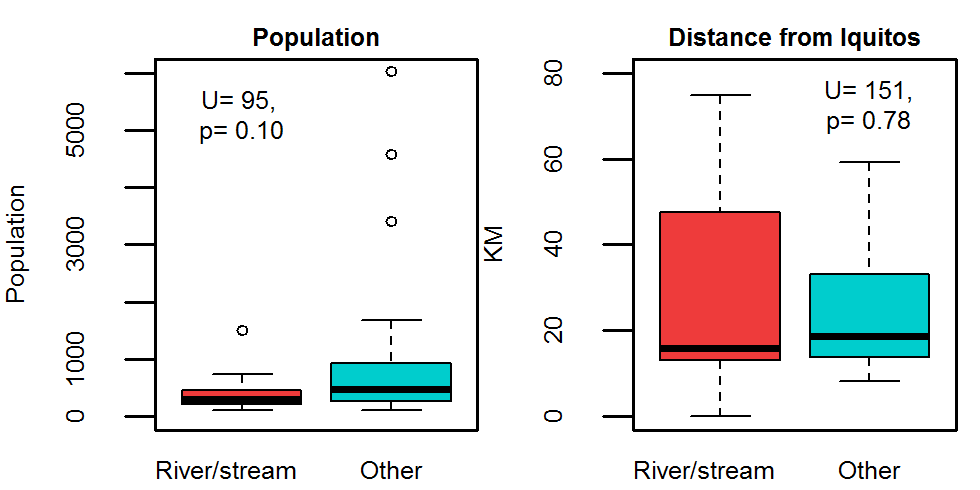

Supplement: Figure S1 — River/stream water vs. other water types by population and distance from Iquitos. Mann-Whitney Wilcoxon tests showed no significant correlation between river/stream water usage and population size or distance to Iquitos. (TIF) [file pntd.0003033.s001.tif]
