# Supplementary material for: Patterns of Geographic Expansion of Aedes aegypti in the Peruvian Amazon
Source: PLoS Negl Trop Dis. 2014 Aug 7;8(8):e3033. doi: 10.1371/journal.pntd.0003033 (PMC4125293; doi:10.1371/journal.pntd.0003033)
Supplement: Table S1 — Characteristics of communities included in the study. + indicates that Ae. aegypti was found, - indicates that Ae. aegypti was not found. A blank space indicates that data were not collected for that community. (DOCX) [file pntd.0003033.s002.docx]

**Table S1. Characteristics of communities included in the study**. + indicates that *Ae. aegypti* was found, - indicates that *Ae. aegypti* was not found. A blank space indicates that data were not collected for that community.

| **Community** | **Houses** | **Inhabitants** | **Year of incorporation** | **Data Source=MOH** | **Data Source=NAMRU** | **Data Source=Collected** | **Year(s) of collections** |
| --- | --- | --- | --- | --- | --- | --- | --- |
| Iquitos | ~81923* | 406340* | 1866 | + | + |  | Ongoing |
| Nauta | 2741 | 13983 | 1830 | + | + |  | Ongoing |
| El Terminal | 1444*** | 6036*** | 1866 |  |  | + | 2012 |
| Tamshiyacu | 1039 | 4583 | 1883 |  | + | + | 2008, 2012 |
| Indiana | 748 | 3410 | 1948 | + | + | + | 2008, 2012 |
| Mazan | 653 | 3184 | 1943 | + | + |  | 2008, 2010, 2011, 2012 |
| Santa Clara de Nanay | 625 | 2868 | 1933 | + |  |  | 2011 |
| Rumococha | 518* | 4341* | 1912 | + |  |  | 2011 |
| Los Delfines | 459 | 1679 | 2000 | + |  | + | 2011, 2012 |
| Quistococha | 402 | 1496 | 1909 | + |  | + | 2011, 2012 |
| Varillal | 371* | 1050* | 1949 | + |  | + | 2012 |
| Padre Cocha | 323 | 1627 | 1917 | + | + |  | 2011 |
| Santo Tomás | 302 | 1308 | 1866 | + |  |  | 2011 |
| 31 de Mayo | 287** | 600** | 2004 | + |  |  | 2011 |
| Cahuide | 167 | 703 | 1965 |  |  | - | 2012 |
| Barrio Florida | 157 | 737 | 1953 | + | + | + | 2011, 2012 |
| Zungaro Cocha | 155 | 782 | 1965 | - | - |  | 2011 |
| Aucayo | 153 | 806 | 1922 | + | + | + | 2008, 2012 |
| Nina Rumi | 139 | 561 | 1928 | - | - |  | 2011 |
| Manacamiri | 136 | 682 | 1913 | - |  |  | 2008 |
| 13 de Febrero | 131 | 473 | 1990 |  | - | - | 2008 |
| Nuevo Horizonte | 131 | 452 | 1985 |  |  | - | 2012 |
| Cruz del Sur | 128 | 455 | 1987 | + |  | + | 2011, 2012 |
| Gallito | 125 | 673 | 1936 |  | - | - | 2008, 2012 |
| Santa María de Ojeal | 121 | 550 | 1928 | + |  | - | 2011, 2012 |
| Peña Negra | 121 | 520 | 1942 | + |  | + | 2011, 2012 |
| San Lucas | 116 | 450 | 1991 |  |  | - | 2012 |
| Ex-Petroleros | 111 | 412 | 1986 |  |  | - | 2012 |
| Santa Clara de Ojeal | 106^*^ | 281^*^ | 1952 | + |  | - | 2011, 2012 |
| Sinchicuy | 105 | 449 | 1904 |  |  | - | 2012 |
| El Dorado | 97 | 312 | 1999 | - |  | - | 2011 |
| Laguna Azul | 96* | 420* | 2004 | + |  |  | 2011 |
| El Triunfo | 94 | 319 | 1989 |  |  | - | 2012 |
| 25 de Enero | 84 | 364 | 1992 | + |  | + | 2011, 2012 |
| Picuroyacu | 82 | 355 | 1930 | - |  |  | 2011 |
| 1 de Febrero | 76 | 290 | 2004 |  |  | - | 2012 |
| Nuevo Milagro | 68 | 241 | 1996 | - |  |  | 2008 |
| Buen Pastor | 66 | 238 | 1977 |  | - |  | 2008 |
| 12 de Abril | 63 | 198 | 1990 |  |  | - | 2012 |
| Santa Clotilde | 58 | 255 | 1995 |  |  | + | 2012 |
| Nueva Unión | 57 | 197 | 2002 | + |  | + | 2011, 2012 |
| Puerto Alemendras | 54 | 205 | 1917 | - |  |  | 2011 |
| Santa Clara de Ojeal III | 52 | 230 | 1990 |  |  | - | 2012 |
| El Paujil | 50 | 185 | 1985 |  |  | - | 2012 |
| La Habana | 43 | 155 | 1986 |  |  | - | 2012 |
| 5 de Abril | 38** | 180** | 1998 |  |  | + | 2012 |
| San José | 34 | 118 | 1997 | - |  | - | 2011, 2012 |
| Independencia | 32 | 106 | 1995 | + |  | - | 2011, 2012 |
| Nuevo San Juan | 30 | 148 | 2000 |  |  | - | 2012 |
| Lupunillo | 18 | 101 | 1932 |  |  | - | 2012 |

*Source: Government health center (2012)
**Source: Interview with lieutenant governor (2012)
***Source: Number of houses estimated from Google Earth imagery (Iquitos, Peru, lat -3798893° lon -73.308773°, DigitalGlobe, Landsat, US Geological Survey: Google Earth), and population was estimated by calculating the average number of people per home for the other communities (4.18) and extrapolating.
