# Supplementary material for: Patterns of Geographic Expansion of Aedes aegypti in the Peruvian Amazon
Source: PLoS Negl Trop Dis. 2014 Aug 7;8(8):e3033. doi: 10.1371/journal.pntd.0003033 (PMC4125293; doi:10.1371/journal.pntd.0003033)
Supplement: Table S2 — Datasets, ecological scales, and statistical analyses employed. (DOCX) [file pntd.0003033.s003.docx]

**Table S2. Datasets, ecological scales, and statistical analyses employed.**

|  | **Historical Data (NAMRU + MOH)** | **Collected Data** | | |
| --- | --- | --- | --- | --- |
| **Entomological Data** | *Ae. aegypti* presence-absence | *Ae. aegypti* presence-absence | | |
| **Scale of Data** | Community | Community | House | Container |
| **Analyses** | - Descriptive maps | - Entomological Indices - Mann-Whitney Wilcoxon tests - Logistic regression | - Logistic regression | - Descriptive statistics - Logistic regression |
