# Supplementary material for: Patterns of Geographic Expansion of Aedes aegypti in the Peruvian Amazon
Source: PLoS Negl Trop Dis. 2014 Aug 7;8(8):e3033. doi: 10.1371/journal.pntd.0003033 (PMC4125293; doi:10.1371/journal.pntd.0003033)
Supplement: Table S3 — Community-level univariable logistic regression models. Statistically significant (p<0.05) variables are shown in bold. Variables were included in the multivariate selection process with an entry criterion of p<0.10. (DOCX) [file pntd.0003033.s004.docx]

**Table S3.** **Community-level univariable logistic regression models.** Statistically significant (p<0.05) variables are shown in bold. Variables were included in the multivariate selection process with an entry criterion of p<0.10.

| **Model** | **Variable** | **OR** | **95% CI** | **SE** | **P** | **AIC** |
| --- | --- | --- | --- | --- | --- | --- |
| **1** | **No. wet containers** | **1.032** | **1.00025, 1.064** | **0.016** | **<0.05** | **35.65** |
| **2** | **Population** | **1.0036** | **1.00026, 1.0070** | **0.0017** | **<0.05** | **36.54** |
| 3 | No. rain-filled containers | 1.07 | 0.9952, 1.14 | 0.036 | <0.05 | 36.63 |
| **4** | **Log(Population)** | **5.06** | **1.88, 13.59** | **0.63** | **<0.05** | **38.15** |
| **5** | **Euclidean dist. from IQT (km)** | **0.94** | **0.88, 0.99** | **0.029** | **<0.05** | **41.52** |
| **6** | **Path dist. from IQT (km)** | **0.94** | **0.90, 0.99** | **0.025** | **<0.05** | **42.13** |
| **7** | **River/ stream water (vs. other types)** | **0.18** | **0.038,0.86** | **0.80** | **<0.05** | **44.88** |
| **8** | **Avg. wet containers/ house** | **1.55** | **1.0016, 2.40** | **0.22** | **<0.05** | **45.45** |
| 9 | Age of town (years) | 1.014 | 0.99, 1.04 | 0.011 | >0.1 | 46.61 |
| 10 | Avg. rain-filled containers/ house | 1.60 | 0.83, 3.094 | 0.34 | >0.1 | 47.92 |
| 11 | Potable water (vs. other types) | 3.60 | 0.56, 23.24 | 0.95 | >0.1 | 48.14 |
| 12 | No. vehicles/ month | 0.9989 | 0.99,1.0011 | 0.0011 | >0.1 | 49.06 |
| 13 | No. high-risk vehicles | 0.9984 | 0.9951, 1.0017 | 0.0017 | >0.1 | 49.15 |
| 14 | Presence of competitors | 0.6 | 0.13, 2.71 | 0.76 | >0.1 | 49.62 |
| 15 | Amazon River access (vs. road) | 1.032 | 0.26 , 4.30 | 0.73 | >0.1 | 50.07 |
