# Supplementary material for: Patterns of Geographic Expansion of Aedes aegypti in the Peruvian Amazon
Source: PLoS Negl Trop Dis. 2014 Aug 7;8(8):e3033. doi: 10.1371/journal.pntd.0003033 (PMC4125293; doi:10.1371/journal.pntd.0003033)
Supplement: Table S4 — House-level univariable logistic regression models. Statistically significant (p<0.05) variables are shown in bold. Variables were included in the multivariate selection process with an entry criterion of p<0.10. (DOCX) [file pntd.0003033.s005.docx]

**Table S4. House-level univariable logistic regression models.** Statistically significant (p<0.05) variables are shown in bold. Variables were included in the multivariate selection process with an entry criterion of p<0.10.

| **Model** | **Variable** | **OR** | **95% CI** | **SE** | **P** | **AIC** |
| --- | --- | --- | --- | --- | --- | --- |
| **1** | **No. competitor mosquitoes present** | **5.44** | **2.64, 11.96** | **0.38** | **<0.001** | **354.50** |
| **2** | **Presence of competitors** | **5.85** | **2.68, 13.17** | **0.403** | **<0.001** | **357.10** |
| **3** | **No. rain-filled containers** | **1.17** | **1.083, 1.27** | **0.0404** | **<0.001** | **360.50** |
| **4** | **No. wet containers** | **1.053** | **1.0061, 1.103** | **0.023** | **<0.05** | **371.40** |
| 5 | Inhabitants/ household | 1.12 | 0.99, 1.25 | 0.057 | >0.05 | 372.60 |
