# Supplementary material for: Patterns of Geographic Expansion of Aedes aegypti in the Peruvian Amazon
Source: PLoS Negl Trop Dis. 2014 Aug 7;8(8):e3033. doi: 10.1371/journal.pntd.0003033 (PMC4125293; doi:10.1371/journal.pntd.0003033)
Supplement: Table S5 — Container-level univariable logistic regression models. Statistically significant (p<0.05) variables are shown in bold. Variables were included in the multivariate selection process with an entry criterion of p<0.10. (DOCX) [file pntd.0003033.s006.docx]

**Table S5. Container-level univariable logistic regression models.** Statistically significant (p<0.05) variables are shown in bold. Variables were included in the multivariate selection process with an entry criterion of p<0.10.

| **Model** | **Variable** | **OR** | **95% CI** | **SE** | **P** | **AIC** |
| --- | --- | --- | --- | --- | --- | --- |
| 1 | Fill method = rain | 0.092 | 0.0036, 2.33 | 1.418 | >0.05 | 997.36 |
| **2** | **Presence of competitors** | **12.60** | **6.96, 22.09** | **0.29** | **<0.001** | **1035.50** |
| **3** | **Type = drum/ tank** | **4.04** | **2.70, 5.95** | **0.20** | **<0.001** | **1048.20** |
| **4** | **Container lid = yes** | **0.15** | **0.060, 0.32** | **0.42** | **<0.01** | **1053.00** |
| **5** | **Solar exposure = yes** | **2.42** | **1.65, 3.64** | **0.20** | **<0.001** | **1066.60** |
| **6** | **Type = plastic container** | **0.44** | **0.31, 0.63** | **0.18** | **<0.001** | **1068** |
| **7** | **Type = toilet/ drain** | **5.24** | **1.50,14.19** | **0.56** | **<0.01** | **1075.30** |
| **8** | **Type = tire** | **3.64** | **1.23, 8.68** | **0.49** | **<0.01** | **1082.80** |
| 9 | Type = animal watering pan | 1.67 | 0.69, 3.43 | 0.40 | >0.10 | 1086.60 |
| 10 | Type = nontraditional | 1.071 | 0.17, 3.52 | 0.73 | >0.10 | 1088 |
| 11 | Material = Metal | 1.40 | 0.80, 2.30 | 0.27 | >0.10 | 1093.40 |
| 12 | Material = Plant | 1.14 | 0.063, 5.51 | 1.027 | >0.10 | 1094.80 |
