# Supplementary material for: Patterns of Geographic Expansion of Aedes aegypti in the Peruvian Amazon
Source: PLoS Negl Trop Dis. 2014 Aug 7;8(8):e3033. doi: 10.1371/journal.pntd.0003033 (PMC4125293; doi:10.1371/journal.pntd.0003033)
Supplement: Table S6 — Container-level univariable logistic regression models demonstrating multicollinearity among predictor variables. The tables below demonstrate logistic regression models using all possible combinations of predictor variables. Significant (p<0.05) predictor variables are shown in bold. (DOCX) [file pntd.0003033.s007.docx]

**Table S6. Container-level univariable logistic regression models demonstrating multicollinearity among all possible combinations of predictor variables.** The tables below demonstrate logistic regression models using all possible combinations of predictor variables. Significant (p<0.05) predictor variables are shown in bold.

| **A** | **Outcome: Presence of competitors** | | | | | |
| --- | --- | --- | --- | --- | --- | --- |
| **Model** | **Variable** | **OR** | **95% CI** | **SE** | **P** | **AIC** |
| **1** | **Container lid = yes** | **0.17** | **0.041, 0.45** | **0.59** | **<0.005** | **593.55** |
| **2** | **Type = plastic container** | **0.41** | **0.24, 0.68** | **0.26** | **<0.001** | **597.41** |
| **3** | **Type =drum/tank** | **2.60** | **1.37, 4.65** | **0.31** | **<0.005** | **601.30** |
| **4** | **Solar Exposure = yes** | **2.11** | **1.23, 3.77** | **0.28** | **<0.01** | **601.71** |
| **5** | **Type = tire** | **6.29** | **1.84, 16.42** | **0.54** | **<0.001** | **601.80** |
| 6 | Type = toilet/drain | 5.089 | 0.80, 17.90 | 0.75 | <0.05 | 606.16 |

| **B** | **Outcome: drum/ tank** | | | | | |
| --- | --- | --- | --- | --- | --- | --- |
| **Model** | **Variable** | **OR** | **95% CI** | **SE** | **P** | **AIC** |
| 1 | Type = plastic container | 3.12 *10^-09^ | 3.32*10^-84^,1.38*10^-138^ | 399.066 | >0.05 | 1474.00 |
| **2** | **Container lid = yes** | **1.68** | **1.31, 2.14** | **0.12** | **<0.0001** | **2162.40** |
| 3 | Type = tire | 5.37*10^-07^ | 9.74*10^-60^ ,18.56 | 384.23 | >0.05 | 2170.20 |
| 4 | Type = toilet/ drain | 1.47*10^-06^ | NA, 26.62 | 303.47 | >0.05 | 2173.70 |
| 5 | Solar Exposure = yes | 0.88 | 0.70, 1.10 | 0.11 | >0.10 | 2177.70 |

| **C** | **Outcome: Container lid** | | | | | |
| --- | --- | --- | --- | --- | --- | --- |
| **Model** | **Variable** | **OR** | **95% CI** | **SE** | **P** | **AIC** |
| **1** | **Solar Exposure = yes** | **0.049** | **0.038, 0.064** | **0.13** | **<0.0001** | **2654.70** |
| **2** | **Type = plastic container** | **2.41** | **2.0045, 2.90** | **0.09** | **<0.0001** | **3417.80** |
| 3 | Type = tire | 5.64*10^-07^ | 5.90*10^-39^, 0.021 | 233.0501 | >0.05 | 3491.40 |
| 4 | Type = toilet/ drain | 0.15 | 0.0083, 0.71 | 1.023 | >0.05 | 3505.90 |

| **D** | **Outcome: Solar exposure** | | | | | |
| --- | --- | --- | --- | --- | --- | --- |
| **Model** | **Variable** | **OR** | **95% CI** | **SE** | **P** | **AIC** |
| **1** | **Type = plastic container** | **0.38** | **0.33, 0.44** | **0.075** | **<0.0001** | **4300.30** |
| **2** | **Type = toilet/ drain** | **4.18** | **1.57, 14.45** | **0.55** | **<0.01** | **4465.10** |
| **3** | **Type = tire** | **6.037** | **2.58, 17.65** | **0.48** | **<0.0005** | **4453.20** |

| **E** | **Outcome: Plastic container** | | | | | |
| --- | --- | --- | --- | --- | --- | --- |
| **Model** | **Variable** | **OR** | **95% CI** | **SE** | **P** | **AIC** |
| 1 | Type = Toilet/ drain | 2.96*10^-07^ | NA, 0.0075 | 184.065 | >0.05 | 4286.60 |
| 2 | Type = tire | 1.075*10^-07^ | 1.13*10^-39^, 0.0040 | 233.05 | >0.05 | 4256 |

| **F** | **Outcome: Toilet/ drain** | | | | | |
| --- | --- | --- | --- | --- | --- | --- |
| **Model** | **Variable** | **OR** | **95% CI** | **SE** | **P** | **AIC** |
| 1 | Type = tire | 1.19*10^-06^ | NA, 7.73 | 1044.46 | >0.05 | 276.81 |
